# In Vivo Antihypertensive and Ex Vivo Vasodilatory Studies of Taxifolin

**DOI:** 10.3390/ph18091420

**Published:** 2025-09-21

**Authors:** Xuye Wang, Xiangyang Xu, Wan Yin Tew, Liyun Ouyang, Xiaoning Yang, Hui Wei Loh, Wen Xu, Wei Xu, Mun Fei Yam

**Affiliations:** 1 Department of Pharmacology, School of Pharmaceutical Sciences, Universiti Sains Malaysia, Pulau Pinang 11800, Malaysia; wangxuye@student.usm.my (X.W.); xiangyang91@stuent.usm.my (X.X.); wytew0507@gmail.com (W.Y.T.); ouyangliyun@student.usm.my (L.O.); yang10062922@foxmail.com (X.Y.); huiwei1506@gmail.com (H.W.L.); 2 College of Pharmacy, Fujian University of Traditional Chinese Medicine, 1 Qiuyang Road, Shangjie, Minhou, Fuzhou 350122, China; 2012029@fjtcm.edu.cn

**Keywords:** hypertension, Taxifolin, nitric oxide (NO), endothelial function

## Abstract

**Background**: Hypertension is a leading cause of cardiovascular morbidity and mortality. Taxifolin has shown cardiovascular benefits, but its antihypertensive mechanisms remain poorly defined. This study aimed to comprehensively elucidate the molecular mechanisms underlying Taxifolin’s blood pressure-lowering effects by integrating network pharmacology, molecular docking, ex vivo functional studies, and in vivo validation. **Methods**: Network pharmacology and molecular docking prioritized targets. Ex vivo thoracic aortas were obtained from healthy male Sprague–Dawley (SD) rats, and rings (3–4 mm) were prepared for vasorelaxation studies. Pathway-specific inhibitors, Western blotting, and ELISA were used to investigate mechanisms. In vivo, spontaneously hypertensive rats (SHRs) received oral Taxifolin 15, 30, or 60 mg/kg once daily for 28 days; propranolol (80 mg/kg) served as the positive control. **Results**: Taxifolin produced robust vasorelaxation in endothelium-intact rings (Rmax ≈ 121%), falling to ~72% after denudation. Relaxation was attenuated by LY294002, ODQ, indomethacin, and glibenclamide. In SHR aorta, Taxifolin increased NO by ~132% and cGMP by ~1.9-fold and upregulated p-Akt and eNOS; LY294002 abolished these effects. In vivo, Taxifolin reduced systolic blood pressure by ≈60 mmHg without adverse changes in hematology, biochemistry, or body weight. **Conclusions**: Taxifolin lowers blood pressure through multiple vascular mechanisms consistent with PI3K/Akt/eNOS, NO–sGC–cGMP, COX-2/PGI_2_ and calcium-handling pathways, supporting its potential as a safe antihypertensive candidate.

## 1. Introduction

Hypertension, a leading risk factor for cardiovascular diseases (CVDs), affects over 1.28 billion people worldwide, with a notable prevalence in middle-aged and elderly populations [1,2]. In Malaysia, the prevalence of hypertension is approximately 30%, and it increases markedly with age [3]. Despite the availability of antihypertensive therapies, many patients remain inadequately controlled, leading to severe complications such as renal failure, stroke, retinopathy, and coronary heart disease [4].

Although current antihypertensive drugs can effectively lower blood pressure, they often cause notable side effects and fail to address key pathological factors such as endothelial dysfunction and vascular remodeling [5]. Impaired vascular tone regulation directly influences blood pressure, organ perfusion, and tissue blood flow, underscoring the need for therapies that can modulate vascular function [6,7,8]. At the endothelial level, PI3K/Akt activates eNOS to generate NO, which subsequently stimulates sGC to increase cGMP, forming an integrated PI3K/Akt/eNOS–NO–sGC–cGMP vasodilatory signaling cascade. In parallel, COX-2-derived prostacyclin (PGI_2_) also contributes to vasodilation and vascular protection through a distinct pathway. These mechanisms play essential roles in regulating vascular tone and maintaining cardiovascular homeostasis [9,10].

Taxifolin (dihydroquercetin), a natural flavanonol found in plants such as Siberian larch and milk thistle seeds, possesses potent antioxidant activity. It exhibits various pharmacological effects, including vasodilatory and antioxidant properties, with minimal toxicity [6,7,8]. By enhancing endothelial function and preserving vascular integrity, this compound shows great therapeutic potential in managing hypertension [11,12]. In addition, Taxifolin has a unique chemical structure characterized by a dihydro-C2–C3 bond, which improves its free radical scavenging capacity and stability compared with other flavonoids. Its molecular formula is C_15_H_12_O_7_, and its chemical structure has been well studied, showing multiple hydroxyl groups that contribute to its strong antioxidant potential [13].

Given the limitations of current antihypertensive therapies and the urgent need for drugs that can both lower blood pressure and protect vascular health, exploring the therapeutic potential of Taxifolin is of significant clinical importance [12,14]. Hypertension is not merely defined by elevated blood pressure but is also characterized by endothelial dysfunction—an early and critical event in the pathogenesis of atherosclerosis and other vascular disorders. The endothelium, which regulates vascular tone through factors such as nitric oxide, becomes impaired under hypertensive conditions, leading to imbalanced vasodilation and increased vascular resistance. Addressing endothelial dysfunction is therefore critical for preventing further cardiovascular damage and improving long-term outcomes [15]. This study seeks to evaluate how Taxifolin regulates vascular tone and improves endothelial function using hypertensive models, aiming to facilitate innovative, safer, and more efficient therapeutic options for managing hypertension, thereby helping to ease the global burden of cardiovascular diseases.

## 2. Result

### 2.1. Network Pharmacology and Molecular Docking Analysis

#### 2.1.1. Screening of Active Components and Targets of Taxifolin

Using the TCMSP database and text mining, seven entries associated with Taxifolin were retrieved. One entry was excluded according to the screening criteria in Section 4, and six entries were kept for subsequent evaluation. Twenty-four predicted targets were obtained from these six entries and standardized using the UniProt database. To identify hypertension-related targets, GeneCards and DrugBank databases were queried using the keyword “hypertension”, retrieving 11,823 disease-associated targets in total. Overlapping targets were identified using a Venn diagram, yielding 17 common targets between Taxifolin and hypertension-related targets.

#### 2.1.2. Construction of Drug–Disease–Target Network

**Figure 1:**
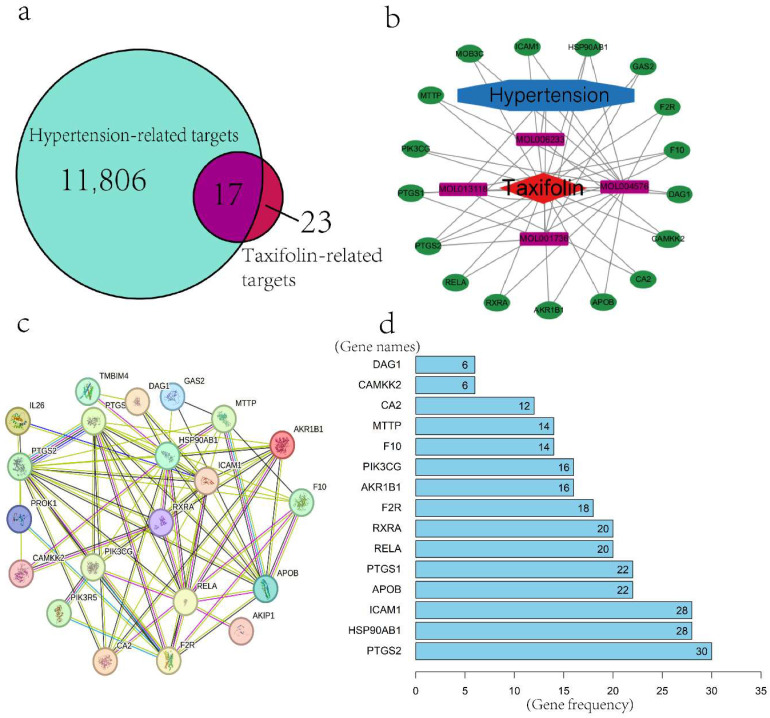
(**a**) Venn diagram with common action targets. (**b**) Network diagram of targets and active ingredients of the Taxifolin-hypertension intersection (Green represents the shared targets between Taxifolin and hypertension, purple represents the four active components of Taxifolin, red represents Taxifolin, and blue represents hypertension. Interactions are indicated by connecting lines. (**c**) PPI network diagram of Taxifolin-hypertension-target. (**d**) Bar chart arranged by the frequency of occurrence of each gene.

By intersecting the Taxifolin targets with hypertension-related targets, 17 common targets were identified and visualized using a Venn diagram (Figure 1a). Cytoscape 3.7.1 was employed to construct a network illustrating the interactions between these 17 shared targets and the six active components of Taxifolin. The final network comprised 23 nodes and 43 edges, highlighting the complex relationships between Taxifolin’s components and hypertension-related targets (Figure 1b).

#### 2.1.3. Protein–Protein Interaction (PPI) Network and Core Gene Screening

To establish the PPI network, the 17 overlapping targets were analyzed via the STRING resource, applying a high-confidence cutoff value greater than 0.90. The network consisted of 21 nodes and 74 connections (Figure 1c). Frequency analysis was performed to identify and rank the top 15 proteins with the highest occurrence within the network, thereby highlighting the core genes potentially involved in Taxifolin’s mechanism of action. The proteins, ranked in descending order of frequency, are: PTGS2, HSP90AB1, ICAM1, APOB, PTGS1, RELA, RXRA, F2R, AKR1B1, PIK3CG, F10, MTTP, CA2, CAMKK2, and DAG1 (Figure 1d).

#### 2.1.4. Gene Ontology (GO) Analysis

**Figure 2:**
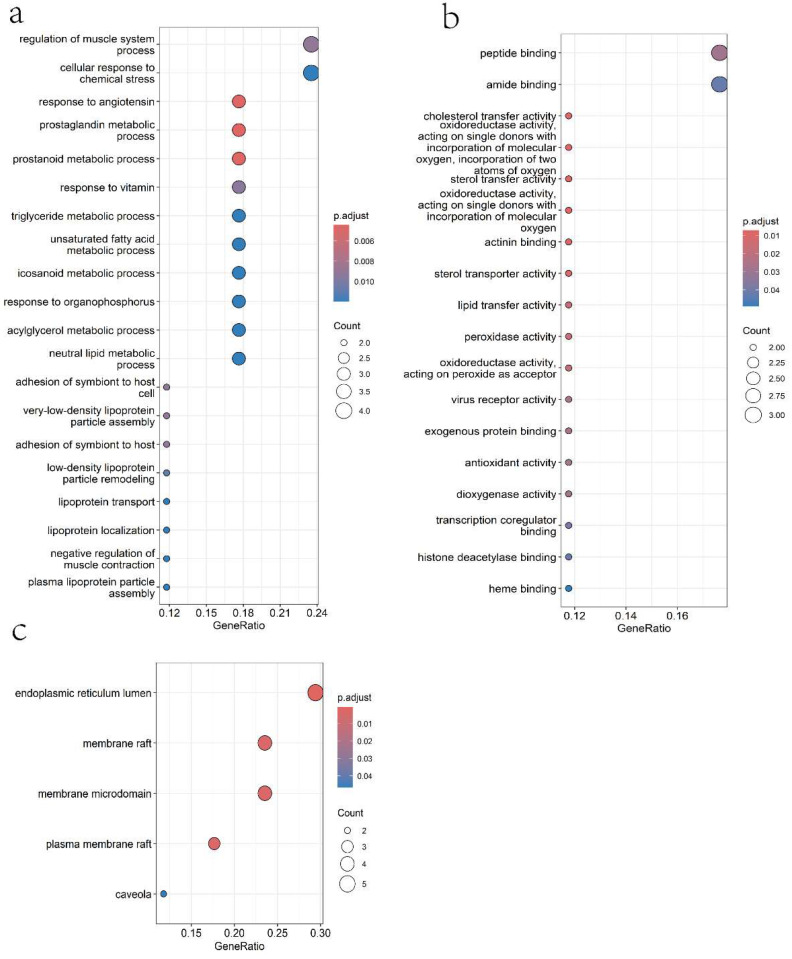
Gene Ontology (GO) enrichment analysis of Taxifolin targets. The top enriched terms in the categories of BP (**a**), MF (**b**), and CC (**c**) are shown. Each bubble represents a GO term, with the color indicating the adjusted *p*-value (red = more significant, blue = less significant). The size of each bubble corresponds to the number of genes associated with that GO term. The *x*-axis shows the gene ratio (number of genes in the term divided by total genes), and the *y*-axis displays the GO term name.

GO enrichment analysis was performed using the clusterProfiler R package (version 4.16.0), focusing on Biological Processes (BP), Cellular Components (CC), and Molecular Functions (MF) with a significance threshold of *p* < 0.05. The targets were primarily associated with: Biological Processes: Response to angiotensin, prostaglandin metabolic processes, very low-density lipoprotein (VLDL) particle assembly, regulation of muscle system processes, triglyceride metabolic processes, and cellular response to chemical stress (Figure 2a). Molecular Functions: Cholesterol transfer activity, oxidoreductase activity, sterol transfer activity, actinin binding, lipid transfer activity, and peroxidase activity (Figure 2b). Cellular Components: Endoplasmic reticulum lumen, membrane rafts, plasma membrane rafts, and caveolae (Figure 2c).

#### 2.1.5. KEGG Pathway Enrichment Analysis

**Figure 3:**
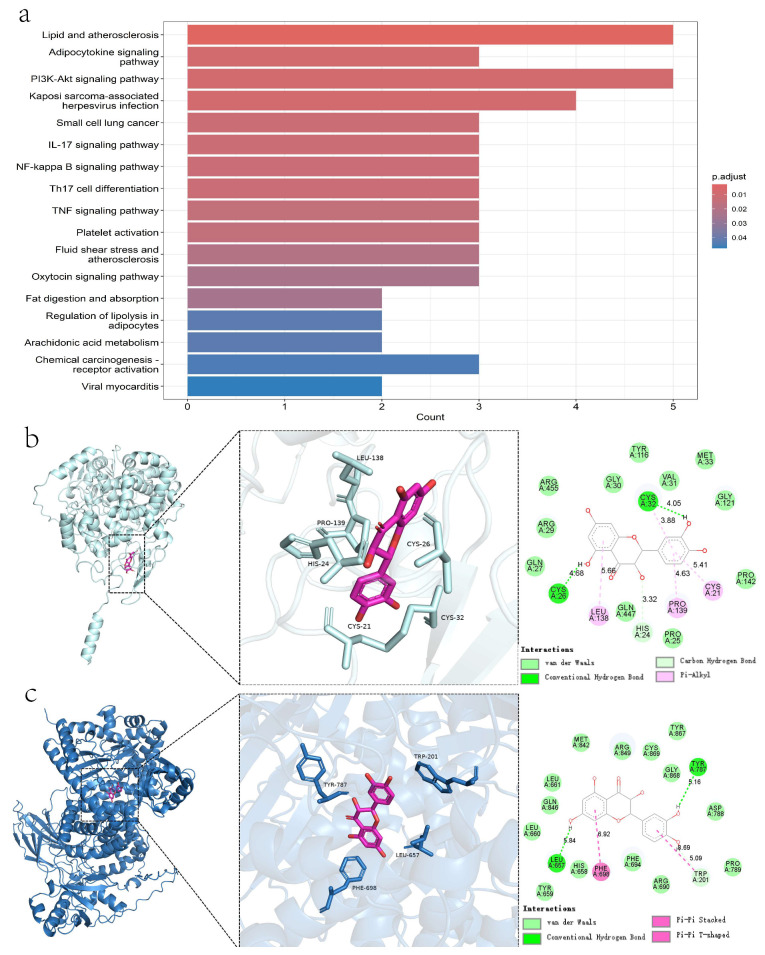
(**a**) Enrichment analysis of KEGG pathway of active components of Taxifolin Decoction in treating hypertension. (**b**) Optimal binding mode of Taxifolin with PIK3CG in the left and schematic diagram of amino acid residues in the active site of PIK3CG in right. Two-dimensional interaction analysis between Taxifolin and PIK3CG protein is below. (**c**) Optimal binding mode of Taxifolin with PTGS2 on the left and schematic diagram of amino acid residues in the active site of PTGS2 on the right. Two-dimensional interaction analysis between Taxifolin and PTGS2 protein is shown below.

Twenty-six pathways were identified as enriched through KEGG analysis and found to be associated with the antihypertensive effects of Taxifolin. The most notable pathways included lipid and atherosclerosis, adipocytokine signaling, PI3K-Akt signaling, NF-kappa B signaling, IL-17 signaling, TNF signaling, platelet activation, and regulation of lipolysis in adipocytes. These pathways were ranked based on ascending *p*-values to highlight their significance (Figure 3a).

#### 2.1.6. Molecular Docking

Molecular docking studies revealed that Taxifolin binds favorably to both PIK3CG and PTGS2, with binding energies of −9.7 kcal/mol and −8.9 kcal/mol, respectively. Two-dimensional interaction analyses indicated that Taxifolin forms van der Waals contacts with surrounding residues in both active sites. The PIK3CG binding pocket is primarily composed of hydrophobic residues (Tyr, Trp, Phe, Leu), while PTGS2 features both hydrophobic (Leu, Pro) and polar (His, Cys) amino acids. These environments underscore the significance of hydrophobic interactions in stabilizing the complexes. Taxifolin also forms hydrogen bonds with Tyr787, Leu657, and Trp201 in PIK3CG, and with Cys26 and Cys32 in PTGS2. In addition, it exhibits π–π interactions with the aromatic residues Phe, Tyr, and Trp in PIK3CG, which may influence fluorescence quenching, and π–alkyl interactions with Leu38, Pro39, and Cys21 in PTGS2. Together, these diverse interactions contribute to the overall specificity and stability of the Taxifolin–protein complexes (Figure 3b,c).

### 2.2. The Vasorelaxant Effects of Taxifolin Were Evaluated Using Isolated Rat Aortic Rings

**Figure 4:**
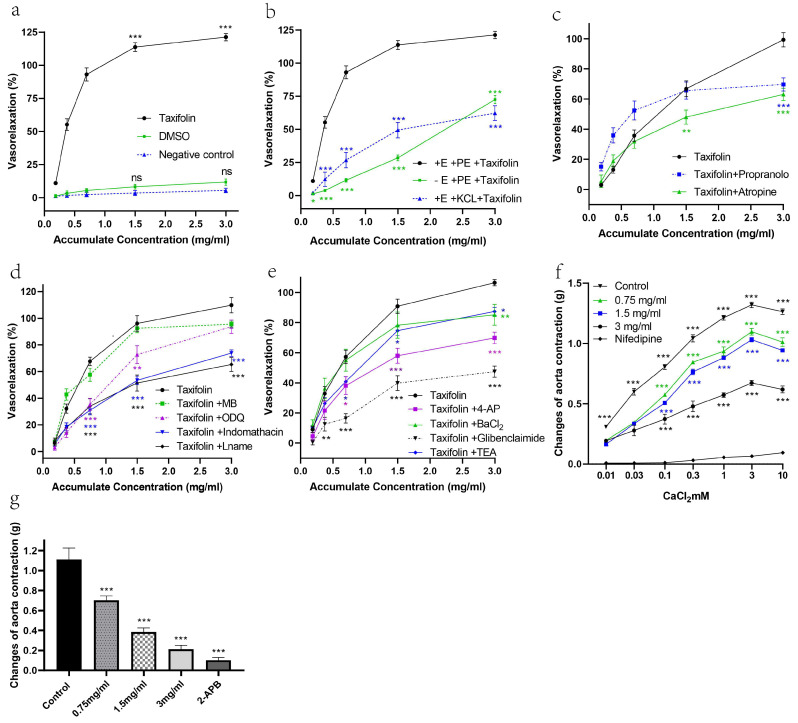
Vasorelaxant effects of Taxifolin on aortic rings under different contractile conditions. (**a**) Vasorelaxation of endothelium-intact rings contracted by KCl. (**b**) Vasorelaxation of endothelium-intact and endothelium-denuded rings contracted by phenylephrine (PE). (**c**) Influence of atropine and propranolol on Taxifolin-induced vasorelaxation in endothelium-intact rings contracted by PE. (**d**) Influence of ODQ, L-NAME, methylene blue, and indomethacin. (**e**) Influence of 4-aminopyridine (4-AP), glibenclamide, tetraethylammonium (TEA), and barium chloride (BaCl_2_). (**f**) Effect of pre-incubation with Taxifolin on CaCl_2_-induced vasoconstriction in endothelium-intact rings under Ca^2+^-free conditions. Negative control received no pre-incubation; positive control was pre-incubated with nifedipine. (**g**) Vasodilative effect of Taxifolin on PE-contracted endothelium-denuded rings under Ca^2+^-free conditions. Negative control received no pre-incubation; positive control was pre-incubated with 2-APB. In all panels, data are expressed as mean ± SEM (n = 6). Symbols *, **, and *** indicate significant differences at *p* < 0.05, *p* < 0.01, and *p* < 0.001, respectively, compared to the corresponding control group.

First, as summarized in Table 1, the vasorelaxant effects of Taxifolin under various treatment conditions are compared. Specifically, Taxifolin induced significant vasorelaxation in arterial rings with intact endothelium. In contrast, neither the DMSO-treated group nor the untreated group exhibited any vasorelaxant effect, indicating that the solvent itself or nonspecific experimental conditions did not interfere with the results (Figure 4a). When the endothelium was removed, the maximum relaxation (Rmax) significantly decreased to 72.47%, suggesting that the endothelium plays a critical role in this process.

Regarding KCl-induced contractions: in arterial rings previously stimulated by KCl, Taxifolin’s relaxation capacity was further diminished, with Rmax decreasing markedly to 62.26%. This result implies the involvement of voltage-dependent calcium channels (Figure 4b).

Furthermore, pretreatment with atropine (a muscarinic receptor antagonist) and propranolol (a β-adrenergic receptor antagonist) led to a further reduction in the vasorelaxant activity of Taxifolin. Specifically, atropine reduced the effect to 63.1%, while propranolol reduced it to 69.7%. These findings indicate that muscarinic receptors and β-adrenergic receptors are involved in the mechanism underlying Taxifolin-induced vasorelaxation (Figure 4c).

For endothelium-dependent assays, aortic rings with intact endothelium were exposed to indomethacin, L-NAME, ODQ, or methylene blue before testing. At a Taxifolin concentration of 1.5 mg/mL, L-NAME and indomethacin markedly reduced vasorelaxation compared with controls, whereas ODQ treatment resulted in only a modest decrease, with relaxation levels of 72.7% and 96.04% in ODQ-treated and control rings, respectively (*p* < 0.01). However, at the concentration of 3 mg/mL, no significant difference was observed. Methylene blue also did not show a significant effect. When examining the vasodilative effects through the endothelium-dependent pathway, the inhibitory effects of the blockers were ranked as follows: L-NAME > indomethacin > ODQ > MB (Figure 4d). This suggests that Taxifolin may exert its vasodilative effect via the NO/PGI2/sGC/cGMP pathway.

Additionally, the influence of K^+^ channel blockers on Taxifolin’s vasorelaxant activity was examined by pre-incubating the aortic rings with TEA, 4-AP, BaCl_2_, and glibenclamide prior to inducing contraction with PE. Among the K^+^ channel blockers tested, TEA and BaCl_2_ showed relatively smaller inhibitory effects on Taxifolin-induced vasorelaxation compared to the other blockers, with relaxation percentages of 87.46%, 85.21%, and 106.5% for the TEA, BaCl_2_, and control groups, respectively (*p* < 0.05). The 4-AP group, however, showed a significant reduction in vasorelaxation compared to the control group, with an Rmax value of 69.84%. Glibenclamide was proven to be the most potent inhibitor, suppressing vasorelaxation by inhibiting the KATP channel. The Rmax value for the glibenclamide group was only 47.44% (Figure 4e).

Additional studies were performed to characterize how Taxifolin regulates intracellular calcium release and extracellular calcium entry. The endothelial integrity of the aortic rings—either preserved or removed—was a key determinant in defining the contribution of calcium channels to Taxifolin-mediated relaxation. Calcium was incrementally added in concentrations ranging from 0.01 to 10 mM to induce a dose-dependent contraction in rings with intact endothelium.

Figure 4f shows that the calcium channel blocker nifedipine (0.01 µM) effectively inhibited contractions induced by Ca^2+^ influx, keeping the contraction force below 0.1 g even at the highest calcium concentration of 10 mM. Taxifolin at 3 mg/mL produced a similar inhibitory effect, while lower concentrations of Taxifolin, such as 0.75 mg/mL, also showed notable effects. In contrast, the untreated control group displayed a maximum contraction of 1.3 g, underscoring Taxifolin’s potential to reduce vascular contraction by inhibiting calcium channels.

Figure 4g presents the results of the intracellular calcium release study, where Taxifolin at concentrations of 0.75, 1.5, and 3 mg/mL significantly suppressed the PE-induced contraction, with contraction values of 0.70 g, 0.39 g, and 0.21 g, respectively (*p* < 0.001). The incubation of 2-APB at 100 µM also suppressed PE-induced aortic contraction compared to the control group. The values are 0.12 g and 1.11 g, respectively, and a *p*-value less than 0.001.

These findings suggest that, in addition to limiting calcium influx through plasma membrane channels, Taxifolin also exerts a direct inhibitory effect on calcium release from the sarcoplasmic reticulum via IP3 receptors (IP3R). This dual mechanism highlights the potential of Taxifolin as a vasodilator, capable of reducing vascular smooth muscle contraction through comprehensive inhibition of calcium channels. An evaluation of Taxifolin’s vasorelaxation potency and peak response was performed across the experimental conditions. For each group, EC_50_ and Rmax values were summarized and compared, as presented in Table 1.

### 2.3. Taxifolin Significantly Increased Nitric Oxide (NO) Levels and Activated Multiple Vasodilatory Pathways in Spontaneously Hypertensive Rats (SHRs)

**Figure 5:**
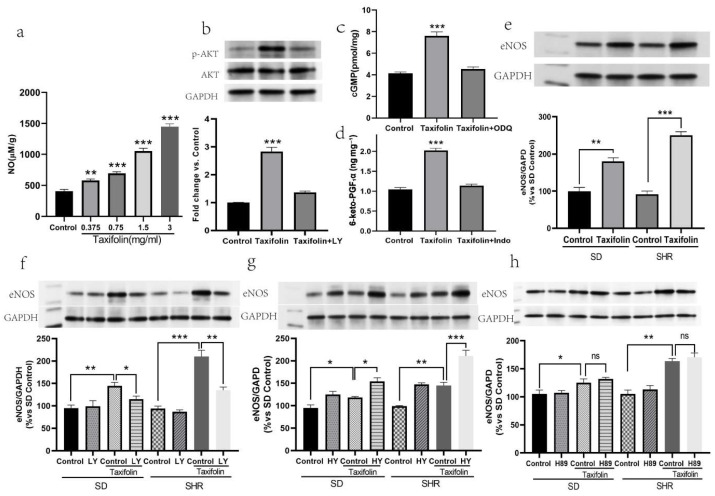
(**a**) Effects of Taxifolin on NO levels in the thoracic aorta of SHR. (**b**) Influence of LY294002 pre-treatment on Taxifolin-induced Akt phosphorylation expression. (**c**) Influence of ODQ pre-treatment on Taxifolin-induced cGMP production. (**d**) Influence of indomethacin pre-treatment on Taxifolin-induced 6-keto-PGF_1_α production. (**e**) Effects of Taxifolin on eNOS expression in the rat thoracic aorta. (**f**) Influence of LY294002 pre-treatment on Taxifolin-induced vasodilation and eNOS expression. (**g**) Influence of HY-N0274 pre-treatment on Taxifolin-induced vasodilation and eNOS expression. (**h**) Influence of H89 pre-treatment on Taxifolin-induced eNOS expression. Symbols *, **, and *** indicate significant differences at *p* < 0.05, *p* < 0.01, and *p* < 0.001, respectively; “ns” indicates no significant difference.

The findings demonstrate that Taxifolin significantly increased the NO levels in the aortic vascular tissues of SHRs (Figure 5a). Incubation of isolated aortic rings with different concentrations of Taxifolin led to a concentration-dependent elevation of NO levels. ELISA results showed that the NO levels in the SHR-control group, SHR-taxifolin (0.75 mg/mL) group, SHR-taxifolin (1.5 mg/mL) group, and SHR-taxifolin (3 mg/mL) group were 405.2 ± 13.31, 579.4 ± 9.97, 694.8 ± 11.01, and 1053 ± 20.10, 1448 ± 21.00 (*p* < 0.05), respectively (Figure 5a). Western blot analysis further confirmed that Taxifolin markedly enhanced endothelial nitric oxide synthase (eNOS) expression in both SD and SHR aortic tissues, with a more pronounced effect in SHRs. The relative band intensity values indicated significant increases in eNOS expression, especially in the SHR-Taxifolin group (*p* < 0.05) (Figure 5e). To explore upstream mechanisms, phospho-Akt (Ser473) levels were measured. Taxifolin treatment significantly increased phospho-Akt by approximately 2.7-fold compared to the control group (*p* < 0.001), without altering total Akt levels. This phosphorylation was completely abolished by LY294002 (10 µM), confirming that Akt activation by Taxifolin is PI3K-dependent (Figure 5b). Taxifolin also led to a significant 1.9-fold increase in cGMP levels (*p* < 0.001), which was entirely reversed by ODQ, supporting the involvement of the canonical NO–sGC–cGMP pathway (Figure 5c). In addition, ELISA results showed that Taxifolin increased 6-keto-PGF1α concentrations by approximately 2-fold (*p* < 0.001), and this effect was completely abolished by indomethacin, confirming activation of the COX-2/PGI2 axis (Figure 5d). Pre-treatment with LY294002 also attenuated eNOS activation, suggesting that the PI3K/Akt pathway regulates eNOS expression in response to Taxifolin (Figure 5f). Moreover, incubation with the NF-κB inhibitor HY-N0274 significantly increased eNOS expression, and Taxifolin further enhanced this effect, indicating that NF-κB inhibition facilitates NO synthesis, synergistically augmented by Taxifolin (Figure 5g). In contrast, pre-treatment with the cAMP/PKA pathway inhibitor H89 did not significantly alter Taxifolin-induced eNOS expression, suggesting that this pathway is not involved in the observed effects (Figure 5h).

### 2.4. In Vivo Antihypertensive Effects of Taxifolin on Spontaneously Hypertensive Rats (SHRs)

#### 2.4.1. Antihypertensive Effect of Taxifolin on Spontaneously Hypertensive Rats

**Figure 6:**
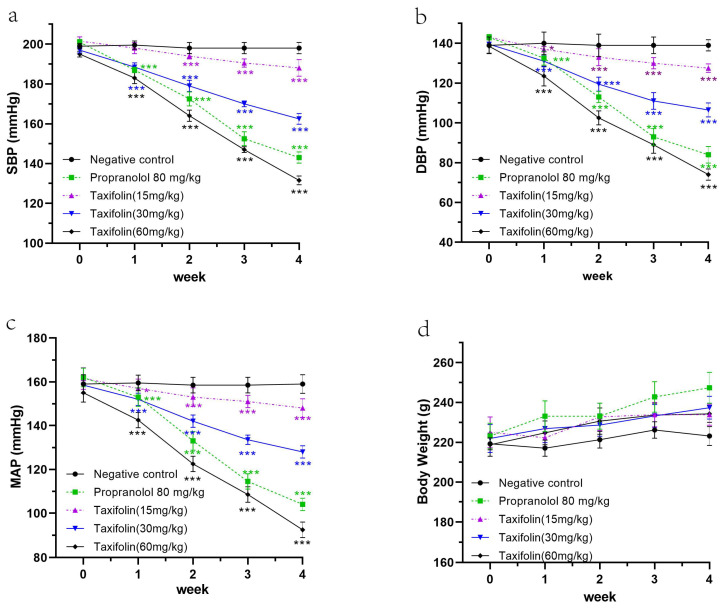
The anti-hypertensive effects of Taxifolin, along with positive and negative controls, on systolic blood pressure (SBP) (**a**), diastolic blood pressure (DBP) (**b**), mean arterial pressure (MAP) (**c**), and body weight (**d**) in SHRs. Significance levels are indicated by * and *** representing *p* < 0.05 and *p* < 0.001, respectively, compared to the negative control group.

In SHRs, Taxifolin administered daily for four weeks dose-dependently reduced SBP, DBP, and MAP. At doses of 15, 30, and 60 mg/kg, the reduction in SBP was 188.34 ± 4.05, 162.25 ± 4.33, and 132.38 ± 3.14 mmHg, respectively. Very similar significant decreases were obtained in both DBP and MAP in comparison to the negative control group. Even at their lowest dose, a meaningful decrease was observed by the fourth week (Figure 6a–c).

#### 2.4.2. Toxicological Evaluation of Taxifolin

Over the 28-day treatment period, Taxifolin showed no adverse effects on the general behavior or activity levels of the SHRs. The rats displayed normal behavior with no signs of distress or lethargy, indicating that Taxifolin did not negatively impact their central nervous system or overall well-being. Body weights remained consistent across all groups, suggesting no interference with metabolic functions or growth (Figure 6d).

No mortalities were recorded at any dose level up to 60 mg/kg, demonstrating good tolerability. Hematological and biochemical parameters—including liver and kidney function assessments—remained within normal reference range. These results indicate that Taxifolin is well-tolerated and safe at the tested doses, supporting its potential as a therapeutic agent for hypertension without significant toxicity (Table 2).

## 3. Discussion

This research thoroughly explored how Taxifolin, a natural flavonoid with strong antioxidant and vascular protective activities, mediates its blood pressure-lowering effects. The antihypertensive effects and underlying mechanisms of Taxifolin were elucidated through network pharmacology, molecular docking, and both ex vivo aortic ring assays and in vivo studies. Two key targets, PIK3CG and PTGS2, were specifically validated based on predictions from network pharmacology. The overall experimental workflow and the key signaling pathways validated are summarized and presented in Figure S1.

Previous studies have demonstrated that PIK3CG and PTGS2 (COX-2) play key roles in endothelial function and cardiovascular homeostasis by modulating inflammatory responses and promoting vasodilation [16,17]. Based on the findings from network pharmacology analysis, these two targets were selected for further molecular docking studies to investigate their potential interactions with Taxifolin. Functional studies revealed that Taxifolin significantly inhibited PE-induced contractions more effectively than KCl-induced contractions. PE acts through the α1 receptor–G protein–IP3 pathway to promote intracellular calcium release, suggesting that Taxifolin acts primarily by limiting calcium mobilization or enhancing endothelial-derived vasodilators (NO/PGI_2_) [18]. In contrast, the reduced effect on KCl-induced contractions, which are mediated by membrane depolarization and voltage-operated calcium channels (VOCCs), suggests a less prominent role for this pathway [19,20].

The central role of eNOS in mediating vasorelaxation was further substantiated by the significant reduction in Taxifolin-induced vasodilation upon eNOS inhibition with L-NAME. Taxifolin also elevated both eNOS expression and NO production. Phosphorylation of Akt at Ser473, which serves as a classical marker of PI3K/Akt pathway activation [21], was markedly increased by Taxifolin and suppressed by LY294002. This indicates that the PI3K/Akt axis serves as a key upstream regulator of eNOS expression and NO synthesis, consistent with previous studies [19,22]. Although phosphorylation at eNOS-Ser1177 was not directly measured, the observed LY294002-sensitive increase in Akt phosphorylation and downstream NO production strongly supports Ser1177 as a likely regulatory site. Phosphorylation at this residue is widely recognized to enhance NO synthesis. Future studies using phosphorylation-specific antibodies targeting eNOS-Ser1177 will help confirm this mechanism [19,23,24,25].

To further support the mechanistic insights obtained from network pharmacology and functional assays, GO enrichment analysis was conducted. Notably, biological process terms such as “response to angiotensin,” “prostaglandin metabolic processes,” and “regulation of muscle system processes” are directly associated with blood pressure control and vascular tone modulation, aligning with findings related to NO and PGI_2_ pathway activation [26,27]. The enrichment of molecular function terms including “oxidoreductase activity” and “peroxidase activity” supports Taxifolin’s antioxidant properties, which may enhance endothelial protection and NO bioavailability. Terms like “cholesterol transfer activity” and “sterol transfer activity” suggest potential involvement in lipid regulation and membrane microdomain dynamics, potentially influencing eNOS signaling [28,29]. Additionally, cellular component terms such as “membrane rafts,” “plasma membrane rafts,” and “caveolae” indicate the importance of specialized membrane domains in mediating Taxifolin-induced effects. These structures serve as signaling hubs for eNOS activation, consistent with the observed PI3K/Akt-dependent eNOS upregulation and enhanced NO/cGMP signaling [30,31]. Overall, the GO enrichment results complement the experimental data by reinforcing the involvement of key pathways and cellular sites in Taxifolin-mediated vasodilation and antihypertensive effects.

It is well established that NO initiates the NO signaling cascade by activating sGC, which catalyzes the conversion of GTP to cGMP. The resulting increase in intracellular cGMP promotes smooth muscle relaxation and vasodilation, positioning this pathway as a central regulator of vascular tone [32]. In this study, activation of this classical NO–sGC–cGMP signaling axis was confirmed, as Taxifolin markedly elevated cGMP levels—an effect completely abolished by ODQ. Furthermore, Taxifolin also increased the production of 6-keto-PGF_1_α, a stable metabolite of prostacyclin (PGI_2_), and this increase was fully suppressed by indomethacin, supporting the concurrent activation of the COX-2/PGI_2_ pathway as a complementary vasodilatory mechanism.

The participation of potassium channels was evident from the strong inhibitory effect of glibenclamide, a K-ATP channel blocker, on Taxifolin-induced vasorelaxation. These channels regulate membrane potential and calcium influx, which are essential for vascular tone modulation [33]. Their involvement supports a multifaceted role of Taxifolin in vascular regulation. Taxifolin also suppressed extracellular calcium influx via VOCCs and intracellular calcium release via IP3 receptors, providing mechanistic insight into its vasorelaxant effect. While L-type calcium channels are likely involved. Clarifying whether a compound acts through direct L-type calcium channel blockade or indirect upstream modulation is crucial for understanding its pharmacological specificity and safety profile [34].

This study also has some limitations. First, although functional assays showed inhibition of Ca^2+^ influx and intracellular release, electrophysiological recordings were not performed. thus, whether Taxifolin acts via direct L-type Ca^2+^-channel block or upstream modulation remains to be clarified. Second, histopathological evaluation was not conducted in this study, although such analysis is critical for confirming the absence of subclinical tissue injury and strengthening the overall safety assessment. Formalin-fixed tissues have been preserved, allowing future blinded histological examination when resources permit. Third, the in vivo evaluation spanned 28 days, which characterizes short-term efficacy and tolerability but does not substitute for longer-term toxicology; extended-duration studies (e.g., 90-day repeated-dose with recovery and comprehensive organ histopathology) are warranted to refine the safety profile [35]. Finally, as DMSO can occasionally induce modest aortic relaxation, we used vehicle-matched controls and observed only a non-significant trend compared with untreated rings (Figure 4a). However, subtle confounding by DMSO cannot be fully excluded. To confirm robustness, future studies will employ lower-DMSO or DMSO-free vehicles, such as HP-β-cyclodextrin, PEG400/Tween-80, or CMC-Na.

## 4. Materials and Methods

### 4.1. Animal

Male Sprague-Dawley (SD) rats weighing 200–240 g were used in the ex vivo aortic ring experiments, while spontaneously hypertensive rats (SHRs) weighing 200–220 g were used in both the in vivo antihypertensive study and the sub-chronic toxicity evaluation. A total of 80 SD rats and 40 SHRs were used in this study. The rats were accommodated in environmentally regulated facilities, where a 12 h photoperiod was maintained, and food and water were available without restriction [36,37].

### 4.2. Network Analysis of Taxifolin’s Potential Mechanisms Against Hypertension

In this study, network pharmacology combined with molecular docking was employed to clarify how Taxifolin may act against hypertension through potential mechanistic pathways. The active constituents of Taxifolin were retrieved from the Traditional Chinese Medicine Systems Pharmacology (TCMSP) platform, followed by screening based on oral bioavailability ≥ 30% and drug-likeness ≥ 0.18 [38,39,40]. Other active components supplemented by SymMap and PubMed were included to ensure comprehensive coverage [41,42].

For these compounds, potential protein targets were extracted from the TCMSP resource and translated into gene symbols via UniProt annotation. To illustrate the compound–target associations, a network framework was established in Cytoscape 3.7.1. Genes related to hypertension were screened from GeneCards and DrugBank databases based on searches using the keyword “hypertension”. Overlapping targets between Taxifolin and hypertension were identified using a Venn diagram generated with ImageGP 1 [43,44].

The STRING resource (interaction confidence > 0.9; organism restricted to Homo sapiens) was utilized to construct a PPI network based on the shared targets, which was subsequently analyzed in Cytoscape using the CytoNCA tool to identify key hub genes through network topology assessment [45,46]. AutoDock Vina 1.1 was employed to conduct molecular docking simulations aimed at predicting the binding interactions between Taxifolin (PubChem CID: 439533) and the key target proteins PIK3CG (Uniprot ID: P48736) and PTGS2 (Uniprot ID: P35354). Protein structures were prepared in PyMol 2.4 by removing water molecules and ligands, adding hydrogens, and generating PDBQT files using AutoDock Tools 1.5.6. Docking parameters were optimized, and binding conformations with the lowest predicted free energies were prioritized [47,48].

The identified targets underwent GO term enrichment and KEGG pathway evaluation, implemented via the R packages clusterProfiler and org.Hs.eg.db. The resulting bubble diagrams depicted key functional roles and signaling cascades relevant to these targets [38,49,50].

### 4.3. Experimental Procedures for Assessing Taxifolin’s Vasorelaxant Mechanisms in Rat Aortic Rings

Chemicals and drugs used in this study included the following, along with their respective functions and suppliers: indomethacin (10 µM, cyclooxygenase inhibitor, Sigma-Aldrich, Burlington, MA, USA), L-NAME (10 µM, nitric oxide synthase inhibitor, Sigma-Aldrich, Burlington, MA, USA), ODQ (10 µM, soluble guanylate cyclase inhibitor, MedChemExpress, Shanghai, China), methylene blue (10 µM, cGMP pathway inhibitor, Sigma-Aldrich, Burlington, MA, USA), atropine (1 µM, muscarinic receptor antagonist, Sigma-Aldrich, Burlington, MA, USA), propranolol hydrochloride (1 µM, β-adrenergic receptor antagonist, MedChemExpress, Shanghai, China), tetraethylammonium chloride (TEA) (1 mM, non-selective calcium-activated K^+^ channel blocker, Sigma-Aldrich, Burlington, MA, USA), 4-aminopyridine (4-AP) (1 mM, voltage-dependent K^+^ channel blocker, MedChemExpress, Shanghai, China), barium chloride (BaCl_2_) (10 µM, inwardly rectifying K^+^ channel blocker, Sigma-Aldrich, Burlington, MA, USA), glibenclamide (10 µM, ATP-sensitive K^+^ channel blocker, MedChemExpress, Shanghai, China), nifedipine (1 µM, MedChemExpress, Shanghai, China), 2-aminoethoxydiphenyl borate (2-APB) (100 µM, IP_3_ receptor inhibitor, Sigma-Aldrich, Burlington, MA, USA), and EGTA (0.2 mM, extracellular calcium chelator, Sigma-Aldrich, Burlington, MA, USA). Other reagents included potassium chloride, dimethyl sulfoxide (DMSO), NaCl, NaHCO_3_, KCl, CaCl_2_, NaH_2_PO_4_, MgSO_4_, and glucose, all from Sigma-Aldrich (Burlington, MA, USA) [51,52,53].

Taxifolin was prepared by dissolving 100 mg in distilled water containing 25% DMSO, with final experimental concentrations ranging from 0.1875 to 3 mg/mL. Fresh Krebs’ solution, prepared separately before every experiment with analytical-grade chemicals, had the following final concentrations: 118.0 mM NaCl, 25.0 mM NaHCO_3_, 4.7 mM KCl, 1.8 mM CaCl_2_, 1.2 mM NaH_2_PO_4_, 1.2 mM MgSO_4_, and 11.0 mM glucose. The solution was continuously aerated and maintained at 37 °C [54,55].

Male Sprague-Dawley (SD) rats weighing 200–240 g were used for ex vivo aortic ring assays. All experiments were independently repeated eight times (n = 8 rings per condition). Rings were randomly assigned to experimental groups for each assay, including initial validation experiments (Taxifolin, vehicle control and blank control), endothelium dependency and calcium channel involvement assays (endothelium-intact PE-precontracted rings, endothelium-denuded PE-precontracted rings, and endothelium-intact KCl-precontracted rings), inhibitor studies (paired control rings without inhibitors and rings pre-incubated with respective inhibitors), and calcium-related experiments [51].

Aortic rings were prepared from rats euthanized by CO_2_ inhalation. After dissection, the thoracic aorta, with all surrounding connective tissues carefully removed, was divided into vascular segments of 2–4 mm. Rings were mounted onto metal wires connected to tension transducers and placed in tissue chambers holding 10 mL Krebs’ solution, temperature controlled at 37 °C and perfused with a gas phase of 95% O_2_ and 5% CO_2_. A 30 min equilibration at a resting load of 1 g was performed, during which the solution was exchanged every 10 min [54,56].

Endothelial integrity was assessed by first contracting rings with phenylephrine (PE, 1 µM) for 10 min and subsequently adding acetylcholine (ACh, 1 µM) for 5 min. Rings showing more than 60% relaxation after ACh administration were regarded as possessing intact endothelium, whereas those with <10% relaxation were classified as endothelium-depleted. Following this assessment, the rings underwent three consecutive rinses in Krebs’ solution freshly prepared for each wash, with 10 min allocated per rinse. This endothelial integrity test was conducted for all rings before proceeding with any experiment [57].

For inhibitor studies, each experiment involved paired groups: control rings (without inhibitor pre-incubation, treated only with Taxifolin) and rings pre-incubated with respective inhibitors for 20 min. Following pre-incubation, rings were contracted with PE (1 µM) and allowed to stabilize for 30 min. Taxifolin was then cumulatively added at 20 min intervals (final concentrations: 0.1875, 0.375, 0.75, 1.5, and 3 mg/mL). Vasorelaxant responses were recorded to evaluate inhibitor modulation effects [58].

Calcium channel experiments involved separate groups: after stabilization in normal Krebs’ solution, the rings were immersed in calcium-depleted Krebs’ buffer to which EGTA (0.2 mM) had been added, followed by a 10 min incubation [59]. Afterward, the rings were washed twice using calcium-depleted Krebs’ buffer without EGTA, each wash lasting 10 min. Rings were pre-incubated with either Taxifolin or nifedipine for 20 min before cumulative addition of CaCl_2_ (0.01–10.0 mM). Contractile responses were recorded to assess calcium influx [60].

Intracellular calcium release experiments utilized endothelium-denuded rings, pre-incubated with either Taxifolin or 2-APB for 20 min prior to PE addition. Contractile responses were measured and compared with untreated control rings [61].

Isometric tension changes were measured using a precision force transducer, amplified by a Quad Bridge Amp, digitized by a PowerLab 26T (ADInstruments, Bella Vista, Australia), and recorded and analyzed using LabChart-5 software. Vasorelaxation was quantified as percentage relaxation relative to the PE-induced contraction using the following formula:

$$Percentage\;of\;relaxation=\frac{\left(B-A)-(C-A\right)}{B-A}\times 100\%$$

where *A* = baseline tension before PE pre-contraction. *B* = plateau contraction after PE pre-contraction. *C* = tension after treatment [62,63].

### 4.4. Ex Vivo Incubation of Rat Aortic Tissues for Mechanistic Analysis of Vasodilatory Signaling Pathways

The experiment of the ex vivo incubation and analysis of rat thoracic aorta tissues was designed to determine the expression level of nitric oxide (NO) and associated signaling proteins. A series of separate experiments were conducted to investigate distinct mechanistic pathways. For the assessment of Akt phosphorylation using Western blot, three experimental groups were formed from the aortic rings: control, Taxifolin (3 mg/mL), and Taxifolin combined with the PI3K inhibitor LY294002 (10 μM). For cGMP quantification by ELISA, three groups were used: control, Taxifolin, and Taxifolin with the sGC inhibitor ODQ (10 μM). For 6-keto-PGF_1_α ELISA analysis, three groups were included: control, Taxifolin, and Taxifolin with the COX inhibitor indomethacin (10 μM). To evaluate eNOS expression under the influence of various signaling inhibitors, three sets of eight-group experiments were conducted using the inhibitors HY-N0274 (NF-κB inhibitor), LY294002 (PI3K inhibitor), and H89 (PKA pathway blocker), each at 10 μM. For each inhibitor, the eight groups were as follows: SD-control, SD-inhibitor alone, SD + Taxifolin, SD + Taxifolin + inhibitor, SHR-control, SHR-inhibitor alone, SHR + Taxifolin, and SHR + Taxifolin + inhibitor. Aortic rings were placed in six-well plates containing 5 mL of Krebs–Henseleit (K-H) buffer and pretreated with the inhibitors at 37 °C for 20 min. For the TAX treatment groups, Taxifolin was introduced to yield a terminal concentration of 3 mg/mL, whereas the control sets were supplemented with 25% DMSO, corresponding to a final concentration of 1.6%. After treatment, the rings were further equilibrated at 37 °C for 5 min.

Protein extraction was carried out by homogenizing vascular tissues in 1 mL of pre-chilled RIPA buffer (Beyotime, Shanghai, China) enriched with 1 mM PMSF and a protease inhibitor mixture. The resulting homogenates underwent ultrasonic disruption on ice at 25% amplitude (three 30 s bursts, interspersed with 30 s pauses) to maximize cell lysis. Following this, the lysates were centrifuged at 12,000× *g* for 20 min at 4 °C, and the clear supernatants were harvested and preserved at −80 °C. Protein concentrations were evaluated at 562 nm using the BCA method (Beyotime, Shanghai, China) calibrated with a BSA reference curve [64,65].

Additional ELISA assays were performed to quantify cGMP and 6-keto-PGF_1_α levels in vascular tissues. For cGMP, an ELISA kit (Cayman Chemical, Ann Arbor, MI, USA) was used following the manufacturer’s protocol. For 6-keto-PGF_1_α, ELISA was performed using a kit from Abcam. Optical density readings were acquired at 450 nm, and the concentrations were estimated through interpolation from the generated standard curves. All samples were run in triplicate, and tissues were stored at −80 °C prior to assay [65].

In a separate experiment, to assess nitric oxide (NO) levels in vascular tissues, SHR aortic rings with intact endothelium were treated with Taxifolin at concentrations of 0.75 mg/mL, 1.5 mg/mL, or 3 mg/mL. Control rings received 10% DMSO. Tissue NO levels were determined using the Griess reaction. Supernatants were collected, and a standard curve was generated with nitrite concentrations of 0, 5, 10, 20, 40, and 80 μM (in triplicate). For NO detection, 100 μL aliquots of each sample were loaded into a 96-well microplate, with three replicates per condition. Afterward, Griess Reagent A (sulfanilamide) and Reagent B (N-1-naphthylethylenediamine dihydrochloride) were applied sequentially, and the wells were incubated in darkness at room temperature for 10 min. Optical density at 550 nm was obtained using a microplate spectrophotometer, and NO concentrations were derived from the generated standard curve [66].

For the Western blot experiment, extracted proteins were mixed with 5× loading buffer (Beyotime) at a 4:1 ratio and denatured at 100 °C for 8 min. Protein extracts were fractionated on 10% SDS–polyacrylamide gels at 80–120 V and subsequently electroblotted onto PVDF membranes using a cooled transfer module operated at 250 mA for 90 min. To minimize nonspecific binding, the membranes were incubated in TBST containing 5% skim milk for 1 h at room temperature, followed by overnight exposure at 4 °C to primary antibodies. The antibodies applied included anti-phospho-Akt (Ser473, 1:1000, Cell Signaling Technology (CST), Danvers, MA, USA), anti-Akt (1:1000, Cell Signaling Technology (CST), Danvers, MA, USA)), anti-eNOS (1:1000, Abcam, Cambridge, UK), and anti-GAPDH (1:3000, Abcam, Cambridge, UK); GAPDH served as the internal normalization control. Upon completion of the rinsing steps, incubation of the membranes with HRP-conjugated secondary antibodies (goat anti-rabbit IgG, 1:5000, Abcam, Cambridge, UK) was carried out for 1 h at room temperature. A chemiluminescence (ECL) imaging system (Thermo Scientific, Waltham, MA, USA) was used to visualize protein signals, and the resulting signals were quantified with ImageJ software (version 1.46r) [66,67].

Data analysis involved comparing NO, cGMP, and 6-keto-PGF_1_α levels across treatment groups, along with protein band intensities for p-Akt, total Akt, and eNOS. This comprehensive analysis elucidated the mechanisms by which Taxifolin regulates vascular relaxation via the PI3K/Akt/eNOS, COX-2/PGI_2_, and NO–sGC–cGMP signaling pathways [68,69].

### 4.5. In Vivo Evaluation of Taxifolin’s Effects and Toxicity in Spontaneously Hypertensive Rats

An in vivo study was conducted to evaluate the effects and potential toxicity of Taxifolin in SHRs. Taxifolin was prepared as a 120 mg/mL stock solution in distilled water containing 25% DMSO and diluted to working concentrations. Propranolol, obtained from MedChemExpress (China), served as a positive control. Healthy male SHRs weighing 200–220 g were allocated into five groups: three groups received oral Taxifolin at doses of 15, 30, or 60 mg/kg, negative control group received 10 mL/kg of the vehicle solution (1.25% DMSO in distilled water), and a positive control group received 80 mg/kg of propranolol in the same vehicle. The animals were dosed by oral gavage once each day over a 28-day treatment period.

Cardiovascular parameters—including systolic blood pressure (SBP), diastolic blood pressure (DBP), and mean arterial pressure (MAP)—were measured weekly using a CODA non-invasive tail-cuff blood pressure monitor. Rats were pre-warmed to 37 °C for 15 min before measurements to enhance detection of tail artery pulsations and improve measurement accuracy. During measurements, rats were placed in restrainers on a temperature-controlled platform maintained at 32–35 °C. Approximately five acclimation cycles were performed first, followed by 10–20 measurement cycles until at least ten valid readings were obtained. Each group consisted of five rats. Body weights were also recorded weekly to monitor overall health and detect any physiological changes due to the treatments. This comprehensive approach allowed for a detailed assessment of Taxifolin’s impact on cardiovascular health over the treatment period [70,71].

After 28 days, a thorough toxicological evaluation was performed. Blood samples were collected via cardiac puncture, obtaining approximately 5 mL from each rat. For biochemical analysis, 3 mL of blood was placed into red-top vacutainers without anticoagulant and allowed to clot. After centrifugation at 4000 rpm for 5 min, serum was separated and stored under controlled conditions for analysis. For hematological analysis, 2 mL of blood was collected into EDTA-containing purple-top vacutainers to prevent clotting and allow complete blood count (CBC) measurement. All analyses were conducted at the Wenzhou BaiYiGe Medical Laboratory Company, an accredited facility with advanced diagnostic equipment. Biochemical parameters were quantified using an Abbott ci2800 analyzer (Abbott Diagnostics, Abbott Park, IL, USA). CBCs were performed using a SYSMEX XE-5000 hematology analyzer (Sysmex Corporation, Kobe, Japan), providing detailed profiles of red and white blood cells and platelet counts. Data were organized using Microsoft Excel 2016 for subsequent statistical evaluation.

### 4.6. Statistical Analysis

All data are presented as mean ± SEM, and statistical evaluations were performed using GraphPad Prism 8.0.

For the 28-day in vivo antihypertensive and body weight studies, two-way repeated measures ANOVA was applied to assess the overall effect of treatment over time. To compare treatment groups at individual time points (e.g., on Day 28), one-way ANOVA was conducted, followed by Dunnett’s post hoc test to compare each treatment group with the negative control.

For other experiments—including ex vivo vasorelaxation assays, ELISA quantification of NO, cGMP, and 6-keto-PGF_1_α, and Western blot analysis—one-way ANOVA with Dunnett’s post hoc test was used. Dunnett’s test was selected because it is appropriate for comparing multiple treatments against a single control while controlling for type I error. Statistical significance was set at *p* < 0.05 [72].

## 5. Conclusions

This study demonstrates that Taxifolin exerts potent antihypertensive effects through multiple synergistic pathways, including PI3K/Akt/eNOS/NO signaling, COX-2/PGI_2_ production, sGC/cGMP signaling, K-ATP channel activation, and modulation of calcium channels. These integrated vascular effects highlight Taxifolin’s promise as a safe and effective therapeutic agent for hypertension, meriting further clinical investigation.

## Figures and Tables

**Table 1:** EC_50_ and Rmax values for Taxifolin-induced vasorelaxation with various antagonists.

Aortic Ring Condition/Antagonist	Taxifolin	
	Rmax (%)	EC_50_ (mg/mL)
KCl-induced vasoconstriction	62.26 ± 5.59 ***	0.58 ± 0.15
Endothelium-intact aortic ring (Taxifolin)	121.2 ± 2.82	0.31 ± 0.01
TEA	87.46 ± 2.76 *	0.48 ± 0.15
Propranolol	69.7 ± 4.30 ***	0.37 ± 0.11
L-NAME	65.33 ± 5.18 ***	0.49 ± 0.17
ODQ	93.62 ± 4.99	0.59 ± 0.16
Indomethacin	73.82 ± 2.45 ***	0.46 ± 0.15
Atropine	63.1 ± 4.10 ***	0.50 ± 0.08
Glibenclamide	47.44 ± 3.71 ***	0.60 ± 0.14
4-AP	69.87 ± 3.81 ***	0.50 ± 0.14
Methylene blue	95.62 ± 2.30	0.44 ± 0.09
BaCl_2_	85.21 ± 5.9 **	0.42 ± 0.24
DMSO	11.75 ± 1.2	N/A
Endothelium-denuded aortic ring	72.47 ± 3.14 ***	1.93 ± 0.02

Notes: EC_50_ represents 50% of effective concentration; Rmax represents maximal relaxation. Results are shown as mean ± SEM, with (n = 8). *, **, and *** show significance levels at *p* < 0.05, 0.01, and 0.001, respectively, in comparison to the control group not pretreated with antagonist.

**Table 2:** Hematology values and Biochemical values of SHRs treated with TAX for 28 days.

			Taxifolin		
Parameters	Negative Control (1.25% DMSO)	Positive Control (Propranolol)	15 mg/kg	30 mg/kg	60 mg/kg
Potassium (mmol/L)	4.72 ± 0.18	4.69 ± 0.18	4.55 ± 0.07	4.63 ± 0.13	4.80 ± 0.19
Platelet Count (×10^9^/L)	860.54 ± 75.32	746.22 ± 82.19	868.36 ± 78.42	839.95 ± 79.21	727.56 ± 81.72
Sodium (mmol/L)	146.13 ± 1.78	146.22 ± 1.72	145.16 ± 1.84	145.39 ± 1.68	145.51 ± 1.67
Chloride (mmol/L)	105.88 ± 1.23	105.65 ± 1.39	105.36 ± 1.26	105.50 ± 1.29	105.97 ± 1.36
Creatinine (µmol/L)	27.23 ± 1.34	26.92 ± 1.21	25.61 ± 1.01	26.25 ± 1.18	27.36 ± 1.29
Blood Urea Nitrogen (mmol/L)	6.10 ± 0.36	6.89 ± 0.39	6.25 ± 0.38	6.60 ± 0.41	5.27 ± 0.37
High-Density Lipoprotein (mmol/L)	1.00 ± 0.11	1.21 ± 0.13	1.09 ± 0.11	1.20 ± 0.11	1.13 ± 0.12
Direct Bilirubin (µmol/L)	0.97 ± 0.08	0.97 ± 0.06	0.87 ± 0.05	0.68 ± 0.05	0.50 ± 0.05
Lymphocyte Percentage (%)	74.12 ± 3.24	72.98 ± 3.64	74.09 ± 3.23	73.27 ± 3.19	73.54 ± 3.34
Aspartate Aminotransferase (U/L)	104.92 ± 11.38	121.45 ± 13.45	115.98 ± 12.98	121.48 ± 11.76	114.90 ± 12.43
Gamma-Glutamyl Transferase (U/L)	3.13 ± 0.50	2.84 ± 0.48	3.21 ± 0.46	3.04 ± 0.49	3.56 ± 0.51
Alkaline Phosphatase (U/L)	100.41 ± 8.29	110.34 ± 9.23	115.42 ± 8.79	109.51 ± 9.06	98.65 ± 9.03
Lactate Dehydrogenase (U/L)	350.15 ± 25.79	338.76 ± 27.12	342.67 ± 24.67	349.21 ± 25.12	358.21 ± 25.87
Triglycerides (mmol/L)	0.75 ± 0.09	0.84 ± 0.08	0.69 ± 0.07	0.82 ± 0.08	0.76 ± 0.09
Cholesterol (mmol/L)	1.51 ± 0.14	1.62 ± 0.15	1.43 ± 0.13	1.49 ± 0.14	1.53 ± 0.14
Low-Density Lipoprotein (mmol/L)	0.51 ± 0.08	0.45 ± 0.09	0.48 ± 0.07	0.54 ± 0.08	0.52 ± 0.08
White Blood Cell Count (×10^9^/L)	8.53 ± 1.02	8.76 ± 1.10	8.91 ± 1.01	8.89 ± 1.08	8.65 ± 1.07
Red Blood Cell Count (×10^12^/L)	8.05 ± 0.54	8.11 ± 0.48	7.85 ± 0.52	7.93 ± 0.53	7.92 ± 0.52
Hemoglobin Concentration (g/L)	151.21 ± 12.52	134.57 ± 11.42	147.28 ± 10.84	148.20 ± 11.32	142.72 ± 10.84
Hematocrit (L/L)	0.42 ± 0.02	0.46 ± 0.01	0.39 ± 0.02	0.41 ± 0.02	0.46 ± 0.01
Mean Corpuscular Volume (fL)	52.31 ± 2.14	56.71 ± 2.03	50.19 ± 1.98	51.04 ± 1.97	57.99 ± 1.87
Mean Platelet Volume (fL)	6.33 ± 0.64	8.41 ± 0.56	6.98 ± 0.59	7.55 ± 0.57	7.15 ± 0.59
Albumin/Globulin Ratio	1.18 ± 0.10	1.50 ± 0.12	1.87 ± 0.13	1.73 ± 0.12	1.80 ± 0.13
Total Protein (g/L)	70.78 ± 5.32	75.81 ± 5.12	66.37 ± 4.89	72.51 ± 5.21	77.71 ± 5.32
Albumin (g/L)	38.5 ± 3.10	45.5 ± 3.01	43.3 ± 3.02	45.9 ± 3.19	49.9 ± 3.27
Globulin (g/L)	33.33 ± 2.45	30.33 ± 2.21	23.1 ± 2.01	26.6 ± 2.04	27.8 ± 2.03
Total Bilirubin (µmol/L)	3.92 ± 0.47	2.25 ± 0.48	2.71 ± 0.42	4.69 ± 0.47	3.81 ± 0.48
Alanine Aminotransferase (U/L)	55.60 ± 5.74	33.51 ± 6.15	42.21 ± 5.81	23.75 ± 5.94	43.13 ± 5.21
Neutrophil Percentage (%)	20.74 ± 2.16	22.14 ± 1.93	21.67 ± 2.24	21.98 ± 2.03	21.76 ± 2.14

## Data Availability

All data generated or analyzed during this study are included in this published article and its Supplementary Materials.

## Supplementary Materials

The following supporting information can be downloaded at: https://www.mdpi.com/article/10.3390/ph18091420/s1, Figure S1: Flow chart of the study.
