# Supplementary figures and images for: In Vivo Antihypertensive and Ex Vivo Vasodilatory Studies of Taxifolin
Source: Pharmaceuticals (Basel). 2025 Sep 21;18(9):1420. doi: 10.3390/ph18091420 (PMC12473947; doi:10.3390/ph18091420)

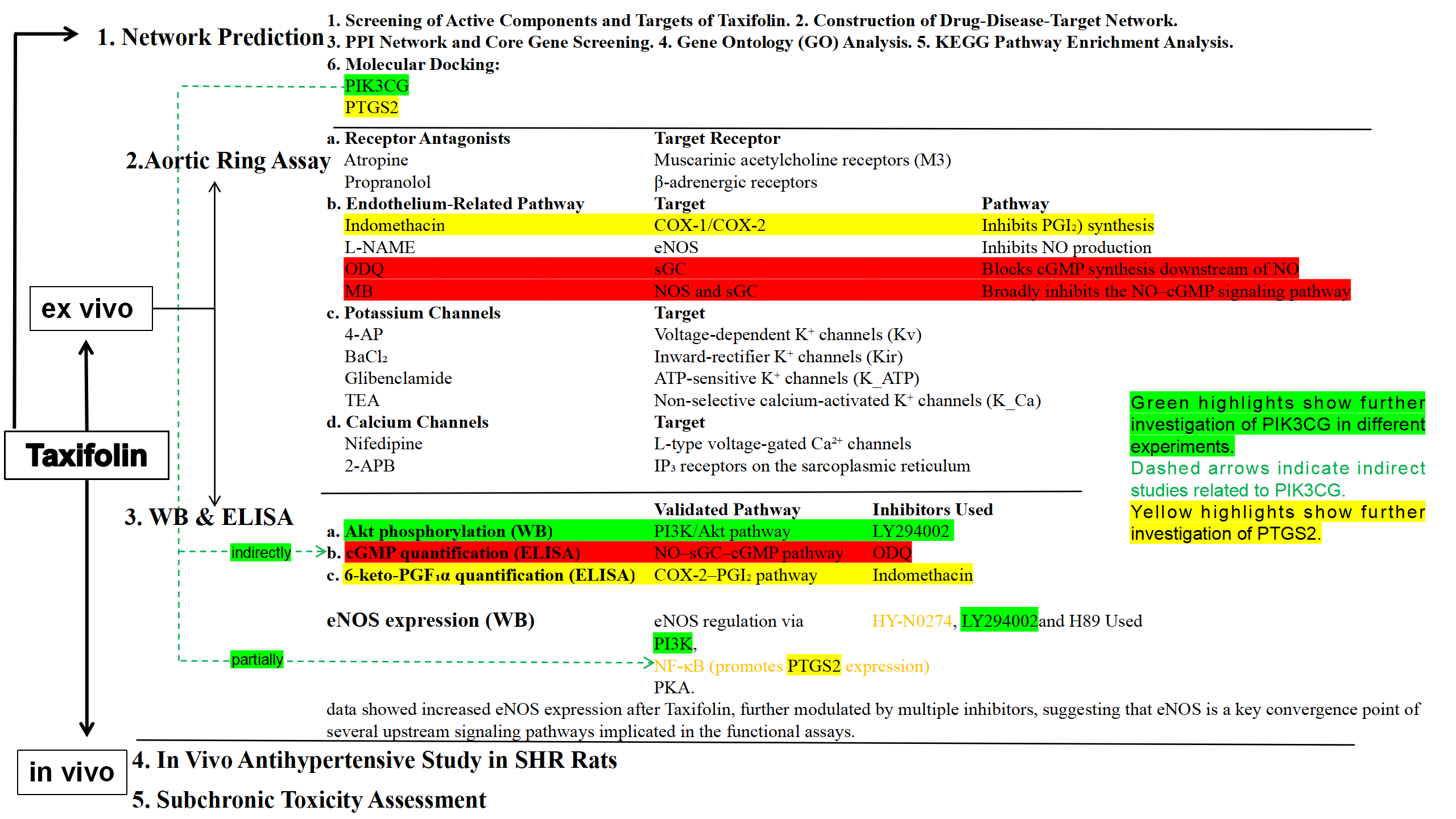

Supplement: Supplementary file 1 [file pharmaceuticals-18-01420-s001.zip › Figure S1. flow chart.tif]
